# Investigating Metal–Insulator Transition and Structural Phase Transformation in the (010)-VO_2_/(001)-YSZ Epitaxial Thin Films

**DOI:** 10.3390/ma11091713

**Published:** 2018-09-13

**Authors:** Yuanjun Yang, Yingxue Yao, Benjian Zhang, Hui Lin, Zhenlin Luo, Chen Gao, Cong Zhang, Chaoyang Kang

**Affiliations:** 1 School of Electronic Science and Applied Physics, Hefei University of Technology, Hefei 230009, China; yaoyingxue@mail.hfut.edu.cn (Y.Y.); zhangbenjian930805@163.com (B.Z.); huilin@hfut.edu.cn (H.L.); 2 National Synchrotron Radiation Laboratory, University of Science and Technology of China, Hefei 230026, China; cgao@ustc.edu.cn; 3 School of Physics and Electronic Information, Henan Polytechnic University, Jiaozuo 454000, China; czhang_94@163.com (C.Z.); kangcy@hpu.edu.cn (C.K.)

**Keywords:** metal–insulator transition, structural phase transition, VO_2_ epitaxial thin film, domain structure

## Abstract

The VO_2_ thin films with sharp metal–insulator transition (MIT) were epitaxially grown on (001)-oriented Yttria-stabilized zirconia substrates (YSZ) using radio-frequency (RF) magnetron sputtering techniques. The MIT and structural phase transition (SPT) were comprehensively investigated under in situ temperature conditions. The amplitude of MIT is in the order of magnitude of 10^4^, and critical temperature is 342 K during the heating cycle. It is interesting that both electron concentration and mobility are changed by two orders of magnitude across the MIT. This research is distinctively different from previous studies, which found that the electron concentration solely contributes to the amplitude of the MIT, although the electron mobility does not. Analysis of the SPT showed that the (010)-VO_2_/(001)-YSZ epitaxial thin film presents a special multi-domain structure, which is probably due to the symmetry matching and lattice mismatch between the VO_2_ and YSZ substrate. The VO_2_ film experiences the SPT from the M1 phase at low temperature to a rutile phase at a high temperature. Moreover, the SPT occurs at the same critical temperature as that of the MIT. This work may shed light on a new MIT behavior and may potentially pave the way for preparing high-quality VO_2_ thin films on cost-effective YSZ substrates for photoelectronic applications.

## 1. Introduction

Vanadium dioxide (VO_2_), which is a typical strongly correlated transition metal oxide, exhibits a first-order metal–insulator transition (MIT) [1,2], which is usually accompanied by a structural phase transition (SPT) from a low-temperature monoclinic semiconductor to a high-temperature rutile metal with a hysteresis of a few kelvins [3]. VO_2_ has the advantages of both reversible and sharp electronic resistivity and optical transmittance changes in the vicinity of MIT (~340 K, near room temperature), which happens in response to different external stimuli, including photons, temperature, electric field, magnetic field, electrical chemistry, and stress [4,5,6,7]. Due to these properties, VO_2_ can be potentially used as a material in numerous applications in electronic and optical devices, such as memory devices [4], Mott field effect transistors [5], thermochromic smart windows [6], and so on.

VO_2_ thin-film structures on the various substrates are still important for the applications in solid-state electronic and optical devices [8,9]. Moreover, the MIT properties can be modulated by substrate engineering; for example, through the proper choice of substrate orientation [10], pre-treatment of the substrate surface [11,12], selection of a substrate type with different lattice parameters [8,13], and so on. Yang et al. observed the anisotropic MIT in the selected (110)-oriented VO_2_/TiO_2_ thin films [10]. Liu et al. used a focused-ion-beam patterning TiO_2_ substrates to control phase separation in the VO_2_ thin films [12]. Then Lee et al. selected the SrTiO_3_, LaAlO_3_ substrates to stabilize the VO_2_ polymorphs [13]. Therefore, significant effort has been exerted into ensuring the successful growth of high-quality VO_2_ epitaxial thin films on certain substrates, such as LaAlO_3_, TbScO_3_, Al_2_O_3_, MgAl_2_O_4_, SrTiO_3_, YSZ, TiO_2_, and so on [8,10,11,14,15,16]. Ramanathan examined the growth of M1 phase VO_2_ thin films on complex oxide single-crystal substrates ((111)-LaAlO_3_, (0001)-Al_2_O_3_, (111)-MgAl_2_O_4_, (111)-MgO) with 3m surface symmetry using RF sputtering techniques [8]. After this, Venkatesan et al. grew high-quality M1 VO_2_ thin films on a SrTiO_3_ substrate by controlling the vanadium arrival rate and oxidation of the V atoms using pulse laser deposition techniques [15]. After those, Lee et al. comprehensively investigated the epitaxial stabilization of M1 VO_2_ on the perovskite-oxide substrates, including TbScO_3_, SrTiO_3_, (LaAlO_3_)_0.3_(SrAl_0.5_Ta_0.5_O_3_)_0.7_, and LaAlO_3_ using pulsed laser epitaxy [13]. Recently, Lee et al. reported that the polymorphic (010)-VO_2_(M1) grew epitaxially on the cubic (001)-YSZ substrate [14]. However, they did not carefully study the MIT behaviors and the SPT in the (010)-VO_2_/(001)-YSZ thin films. So far, there has been a lack of comprehensive investigation into both the MIT and structural phase transformation in this system. On the other hand, from the viewpoint of the engineering applications of the VO_2_ thin films, the wafer-scale YSZ with good optical transmittance is a more cost-effective substrate compared to perovskite oxide single crystals [8,14]. Therefore, combined with the advances made in the RF sputtering techniques, the high-quality VO_2_/YSZ epitaxial thin films in the wafer-scale size may be an attractive candidate material for photoelectronic applications in the future [9,17,18].

Motivated by the above-mentioned progress in the thin-film heterostructures based on VO_2_ and the lack of research on the MIT and the SPT in the new VO_2_/YSZ thin films, we utilized the RF sputtering techniques to grow high-quality (010)-VO_2_/(001)-YSZ thin films in this present study. The MIT behaviors and the SPT were comprehensively studied. The four orders of magnitude of the MIT were achieved, which resulted from the coactions of electron concentration and mobility. The SPT happens synchronously with the occurrence of the MIT. This work potentially provides a way to prepare wafer-scale VO_2_/YSZ thin films and also gives a new insight into the MIT in this system.

## 2. Materials and Methods

### 2.1. Film Fabrication

A RF sputtering technique was employed for growing epitaxial VO_2_ thin film on (001)-oriented Yttria-stabilized zirconia (YSZ, Zr:Y ratio approximately 91:9) substrates. One-side-polished YSZ crystals were commercially bought from MTI Corp. (Hefei, China). A vanadium metal disk of 99.99% purity with a diameter of 2 inches was used as the sputtering target and was water-cooled during deposition. The base pressure of the system was 2.8 × 10^−4^ Pa. The substrate temperature was set to 550 °C. The gas-flow rates of the pure Ar and O_2_ were set to 39 and 8 standard cubic centimeters per minute, respectively. The total pressure for film growth was approximately 0.52 Pa. The sputtering power was chosen as 60 W. The thickness of the VO_2_ thin film in this work was approximately 90 nm and was achieved by controlling the deposition time, which was reported in our previous results [19,20,21].

### 2.2. Structural Characterizations

X-ray diffraction (XRD, Rigaku, Tokyo, Japan) and Raman spectroscopy were employed to investigate microstructural properties. The θ–2θ scans were collected on the X-ray diffractometer with the temperature being varied in situ (Rigaku Smart-lab, Tokyo, Japan, Cu Kα ~ 1.5406 Å). Due to the weak intensity of the asymmetric diffraction peaks, the Φ scans were carried out on the beamline 14B in Shanghai synchrotron radiation facilities (SSRF, Shanghai, China) at room temperature in order to determine the domain structures and epitaxial relationship in the VO_2_ thin films. Raman spectroscopy with an acquisition time of 1 s was conducted using the XploRA^TM^ Raman spectrometer (HORIBA Scientific, Ltd., Paris, France) with in situ controlling temperature across the MIT. A 532-nm laser of 0.25 mW was used as the excitation source with a 100× microscope objective. Each Raman spectrum was repeated three times until the temperature in situ was stable.

### 2.3. Transport Property Measurements

The transport properties were measured on the Hall-effect measurement system (HMS-5500, Ecopia, Korea) using the van der Pauw method. The four Au/Ti (200 nm/20 nm) electrodes were deposited by electron beam evaporation at room temperature. The four probes of the Hall-effect measurement system pressed on the electrodes for the transport measurements.

## 3. Results and Discussion

### 3.1. Phase and Domain Structures in the VO_2_/YSZ Thin Films

**Figure 1:**
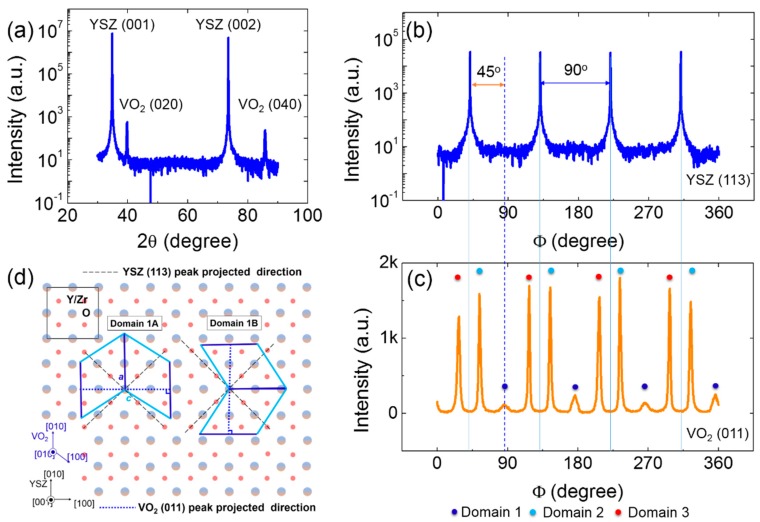
(**a**) XRD θ–2θ scans for the (010)-VO_2_/(001)-YSZ thin films; (**b**) The Φ scan of the substrate YSZ (113) peaks; (**c**) The Φ scan of the VO_2_ (011) peaks; (**d**) The schematic of the domain structures of the VO_2_ epitaxial thin film on the YSZ substrate.

A wide-angle θ–2θ scan of the VO_2_/YSZ thin films was performed at room temperature. The corresponding results are shown in Figure 1a. First, the Bragg’s angles at 39.8° and 85.7° correspond to the (020) and (040) peaks of the VO_2_ thin films, respectively, which are the feature diffraction peaks for the M1 phase of VO_2_ [14,18,22]. The YSZ (001) and (002) peaks are also marked in Figure 1a. The absence of any other peaks of the VO_2_ thin films in the XRD pattern indicates that no other phases of vanadium oxide are present. Hence, the pure M1 VO_2_ thin films were successfully grown on the (001)-YSZ substrate using an RF magnetron sputtering technique. Second, the VO_2_ thin films are highly oriented along the out-of-plane direction with the (010) planes parallel to the (001) surface of the YSZ substrates, which is namely ${\left[010\right]}_{VO_{2}}$//${\left[001\right]}_{\mathrm{YSZ}}$.

To obtain information about the epitaxial relationship of the VO_2_ film with respect to the YSZ substrate, Φ scans were performed on the YSZ (113) and VO_2_ (011) peaks. The four YSZ (113) peaks with the 90° separation are seen in Figure 1b and are marked using solid lines. This fourfold symmetry is definitely ascribed to the nature of the cubic symmetry of the YSZ substrate [14]. Figure 1c shows the Φ scan of the VO_2_ (011) peaks. There are 12 peaks, which are divided into three types of domain structures, which are namely Domain 1 (deep blue dots), Domain 2 (light blue dots), and Domain 3 (red dots), which is shown in Figure 1c. Every domain has four VO_2_ (011) peaks, which are all located at an angle of 90° from each other. This is another of saying that the VO_2_ (011) peaks show fourfold symmetry in the Φ scan. However, it is well-known that the M1 VO_2_ (P2_1_/c space group) must show twofold symmetry in the Φ scan along the rotating axis of [010] direction [23]. It is reasonable that Domain 1 has two different configurations, which has been supported by the findings of Zou and Lee [14,24]. Figure 1d presents the two types of configurations of Domain 1, which are referred to as Domain 1A and 1B. The dashed and dotted lines in Figure 1d show the projected directions of the YSZ (113) and VO_2_ (022) planes projected directions on the surface of the substrates. The two projected directions have an intersection angle of 45°, which is consistent with the Φ scans shown in Figure 1b,c. Hence, the in-plane epitaxial relationship is ${\left[100\right]}_{VO_{2}}$//${\left[010\right]}_{\mathrm{YSZ}}$ in the domain 1A or ${\left[100\right]}_{\mathrm{YSZ}}$ in the Domain 1B. The configurations of Domain 2 and 3 are sketched in the Figures S1 and S2, respectively. The observed domain structure of the (010)-VO_2_/(001)-YSZ epitaxial thin film is quite distinctive compared to the cases in the epitaxial thin film systems of VO_2_/Al_2_O_3_ [24], VO_2_/SrTiO_3_ [25], and VO_2_/TiO_2_ [20]. The multi-domain structures in the VO_2_/YSZ epitaxial thin films are due to the lattice mismatch and surface symmetry match between VO_2_ and the YSZ substrate [14]. This fundamental question will be comprehensively investigated in the future. This striking domain structure may play an important role in the MIT in the VO_2_/YSZ epitaxial thin films.

### 3.2. Studies of Metal–Insulator Transitions

**Figure 2:**
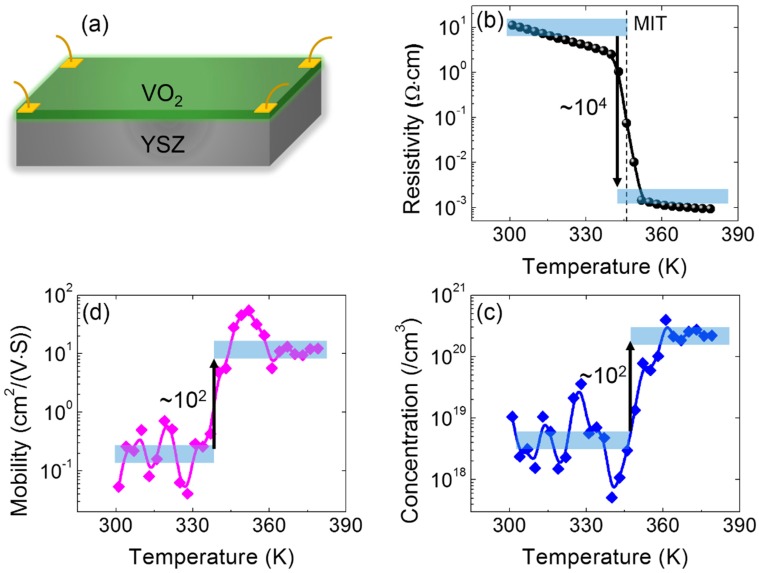
Metal–insulator transition characterizations. (**a**) The schematic of the transport measurement using the van der Pauw method; (**b**) the resistivity vs. temperature curve; (**c**) the electron mobility and (**d**) concentration as temperature increases.

The MIT behaviors were characterized by obtaining the transport properties measurement using the van der Pauw method, which is shown in Figure 2a. The resistivity is a function of heating temperature, as shown in Figure 2b. At 301 and 379 K, the resistivity is 11.1 × 10^−4^ and 9.3 × 10^−4^ Ω·cm, respectively. As a consequence, the amplitude of resistivity change is approximately 1.2 × 10^4^ at the low and high temperature, as shown in Figure 2b. This amplitude of MIT is comparable with the cases of the VO_2_/Al_2_O_3_ and VO_2_/TiO_2_ epitaxial thin films [21,26]. Additionally, the temperature of MIT is about 342 K in the heating cycle, which is consistent with the temperature of 340 K for the bulk VO_2_ [27]. It is known that the VO_2_ is an *n*-type semiconductor in the insulating phase (M1). After the resistivity measurements, the electron concentration and mobility were also measured using the van der Pauw method (please see the detailed procedures in Section 2 of the Supplementary Materials). Both are roughly increased by the order of magnitude of 10^2^ across the MIT, which is shown in Figure 2c,d. It is mentionable that the beating of the mobility and concentration data is due to the displacement of the four probes, resulting from the magnet mechanical movements during measurements (please see Figure S3 in the Supplementary Materials). Therefore, the amplitude of the MIT is about 10^4^, since the resistivity is positively proportional to the product of the electron concentration and mobility. The change in the electron concentration was the sole contribution to the amplitude of the MIT, both in the VO_2_ single crystals [28] and VO_2_/Al_2_O_3_ thin film system [29]. However, in this work, both electron concentration and mobility contribute to the MIT in the VO_2_/YSZ system. This important and interesting observation may encourage the further studies of the MIT mechanism, which may be quite different from the cases in the other VO_2_ film systems. For the mechanisms of this observation, on one hand, the unique domain structure may contribute to the co-actions of the mobility and carrier concentration to the MIT [19]. On the other hand, the oxygen vacancy and defect may play a prominent role in the mobility and carrier concentration across the MIT [30,31]. The detailed studies on these two mechanism are underway and will be present elsewhere.

### 3.3. Investigating Structural Phase Transitions

**Figure 3:**
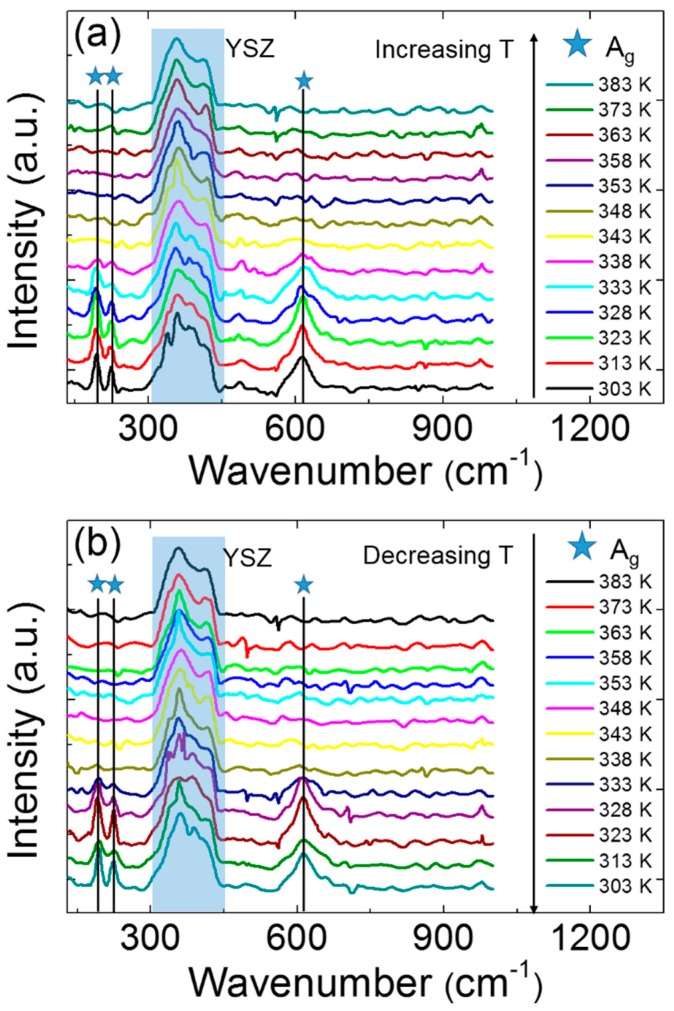
Raman spectra in the cases of increasing (**a**) and decreasing (**b**) temperature. The stars labelled in the figure represent the A_g_ modes in the VO_2_ thin films. The shade boxes show the Raman signals from the YSZ substrates.

Since the SPT often accompanies the MIT [32], the microstructural characterizations were performed using Raman spectroscopy and high-resolution XRD under variable temperature conditions in situ. Figure 3a,b show the Raman spectra during the heating and cooling process. First, the feature Raman peaks at the wavenumbers 192, 223, and 614 cm^−1^ are clearly observed, which were ascribed to the A_g_ modes of the VO_2_ labelled by the stars in Figure 3. This result indicates the phase type of M1 VO_2_. This result is also fairly consistent with the aforementioned XRD patterns. Second, in the heating cycle, the feature Raman peaks disappear at 343 K in Figure 3a, indicating an occurrence of SPT from M1 to the R phase [33]. The SPT temperature is close to that of MIT (~342 K), as determined by transport measurements in Section 3.2. During the cooling process, the feature Raman peaks appear at 338 K, which implies a phase transition from the metallic rutile phase to the insulating M1 phase. The thermal hysteresis (about 5 K) shows a typical first-order phase transition in the (010)-VO_2_/(001)-YSZ epitaxial thin films, which is quite similar to the results of the VO_2_/Al_2_O_3_ [33], VO_2_/TiO_2_ (including the (110)-, (100)-, and (101)-VO_2_/TiO_2_ thin films, whether the thickness thin or thick) [10,20], and VO_2_/SrTiO_3_ thin films [25].

**Figure 4:**
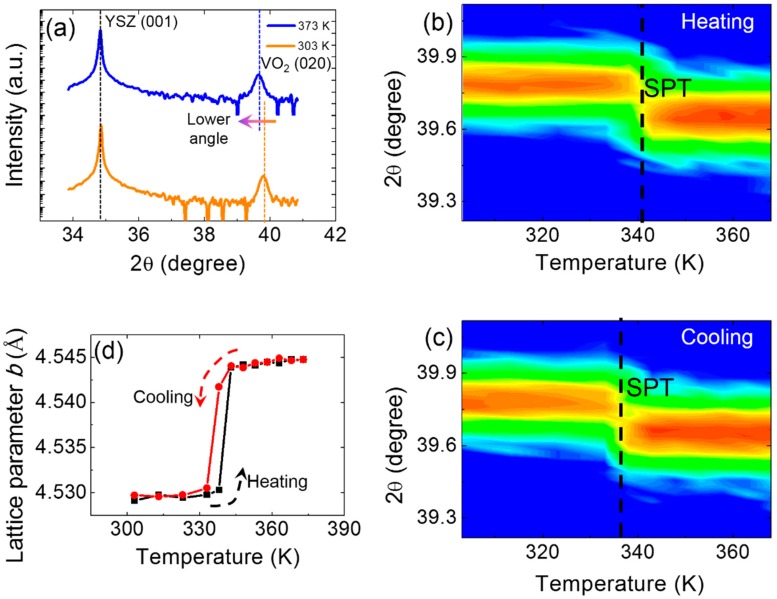
The structural phase transition studied by XRD. (**a**) The local XRD θ–2θ scans of the VO_2_ (020) peaks at 303 and 373 K. The evolutions of the VO_2_ (020) peaks under increasing (**b**) and decreasing (**c**) temperatures. (**d**) The lattice constant *b* vs. the temperature curve.

To further elucidate the SPT across the MIT, the local XRD θ–2θ scans were collected with variable temperatures in situ. In Figure 4a, the (020) peak is shifted down from 39.779° at 303 K to 39.638° at 373 K. Thus, the distance of (020) planes is increased from the low-temperature M1 phase to the high-temperature R phase. To more clearly show the SPT, numerous local θ–2θ scans in the heating and cooling cycles were obtained, which are shown in Figure 4b,c. The sharp shifts of (020) peaks are marked by dashed lines in Figure 4b,c. The SPT temperatures are approximately 343 K and 338 K during the heating and cooling process, respectively. These results are consistent with the transport property measurements and Raman spectra. To quantitatively study the SPT, the lattice constant *b* was measured. First, as increasing and decreasing temperature, the θ–2θ scans of (020) peaks of the VO_2_ thin film were obtained. The *b*-axis is just perpendicular to this characteristic (020) plane. Hence the spacing of the (020) planes directly reflects the lattice constant *b*. Second, the position (2θ) of the (020) peaks can be read out from the θ–2θ scans curves as patterned in Figure 4b,c. Third, using Bragg formula *b*·sin(2θ/2) = λ, the lattice constant *b* can be easily calculated. Consequently, the lattice constant *b* (defined in the notion of monoclinic symmetry) is plotted in Figure 4d. The marked feature was a sharp increase in the lattice constant *b* from 4.529 Å at the low temperature to 4.545 Å at the high temperature, evidencing the occurrence of SPT at 340 K [34]. Therefore, the occurrence of SPT is a natural concomitant of the MIT in the (010)-VO_2_/(001)-YSZ film system. This behavior was also found in the M1 VO_2_/Al_2_O_3_ thin films [31], but was different from the case in the thinner (001)-VO_2_/TiO_2_ film system. In the thinner case, the SPT is suppressed by epitaxial strain, although the MIT continues in the (001)-VO_2_/TiO_2_ thin films [20].

## 4. Conclusions

In summary, the high-quality M1 (010)-VO_2_ thin films were grown epitaxially on the cubic (001)-YSZ substrates. Both the MIT and structural phase transformation were studied. First, the amplitude of the MIT in the VO_2_/YSZ thin films is in the order of magnitude of 10^4^, while the MIT temperature is about 342 K during the heating process. Second, the co-actions of electron concentration and mobility on the MIT in this VO_2_ film system are different from the cases seen in the other system. This result may be due to the distinctive domain structures of the monoclinic VO_2_ thin films on the cubic YSZ substrates. Third, the SPT transforms from the low-temperature M1 phase to the high-temperature rutile phase during the heating process, which was determined by Raman spectroscopy and high-resolution XRD. The temperature of the SPT is nearly same as that of the MIT. Hence, when the VO_2_/YSZ thin films experience the MIT, the SPT will occurs synchronously. This work may highlight another novel VO_2_ thin-film system, which probably has a novel MIT mechanism. Our findings may pave the way to using these thin films on cost-effective substrates in photoelectronic applications.

## Supplementary Materials

The following are available online at http://www.mdpi.com/1996-1944/11/9/1713/s1, Figure S1: Domain configurations of Domain 2; Figure S2: Domain configurations of Domain 3, Figure S3: (a) Ecopia HMS-5000 Hall Measurement System. (b) The four-probe stage with movable magnets.
