# Supplementary material for: Investigating Metal–Insulator Transition and Structural Phase Transformation in the (010)-VO2/(001)-YSZ Epitaxial Thin Films
Source: Materials (Basel). 2018 Sep 13;11(9):1713. doi: 10.3390/ma11091713 (PMC6163228; doi:10.3390/ma11091713)
Supplement: Supplementary file 1 [file materials-11-01713-s001.pdf]

Supplementary Materials: Investigating Metal–Insulator Transition and Structural Phase Transformation in the (010)-VO_2/(001)-YSZ Epitaxial Thin Films

Yuanjun Yang [1,*], Yingxue Yao [1], Benjian Zhang [1], Hui Lin [1], Zhenlin Luo [2,*], Chen Gao [2], Cong Zhang [3] and Chaoyang Kang [3]

1. Domain Configurations of Domain 2 and Domain 3

Domain 2 has two configurations as shown in Figure S1. The projected directions of the VO_2 (011) peaks from Domain 2A and 2B are marked by dotted lines. And those of YSZ (113) peaks are shown by dashed lines. The intersection angels of the projected directions are well consistent with the Φ scans in Figure 1b,c in the main text. Domain 3 also has two similar configurations, Domain 3A and 3B in Figure S2.

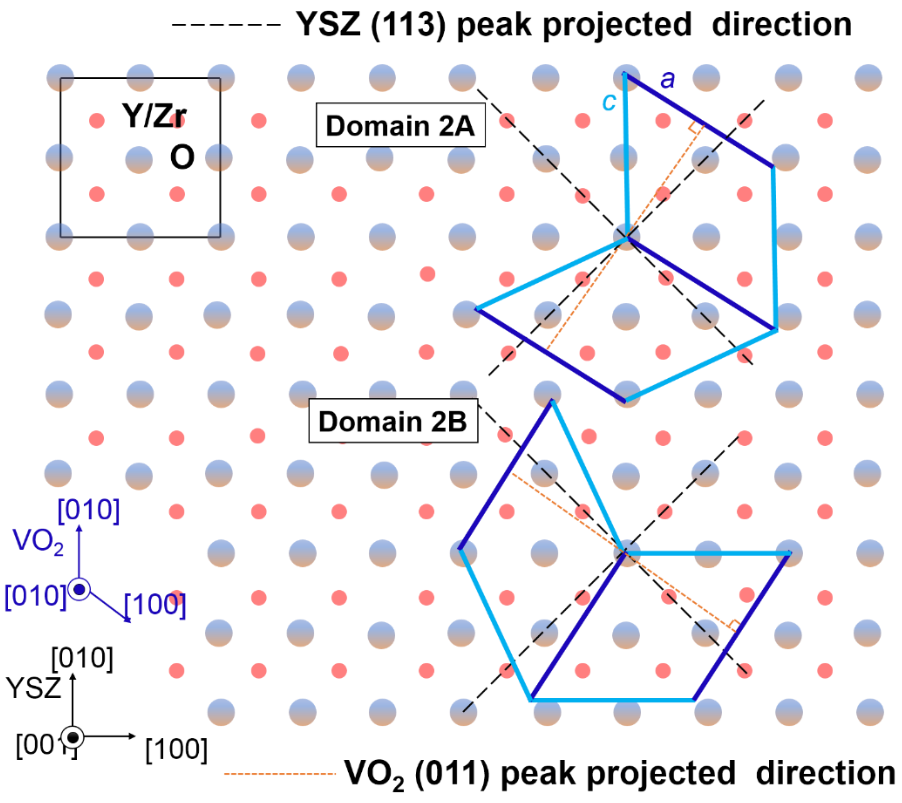

Figure S1. Domain configurations of Domain 2.

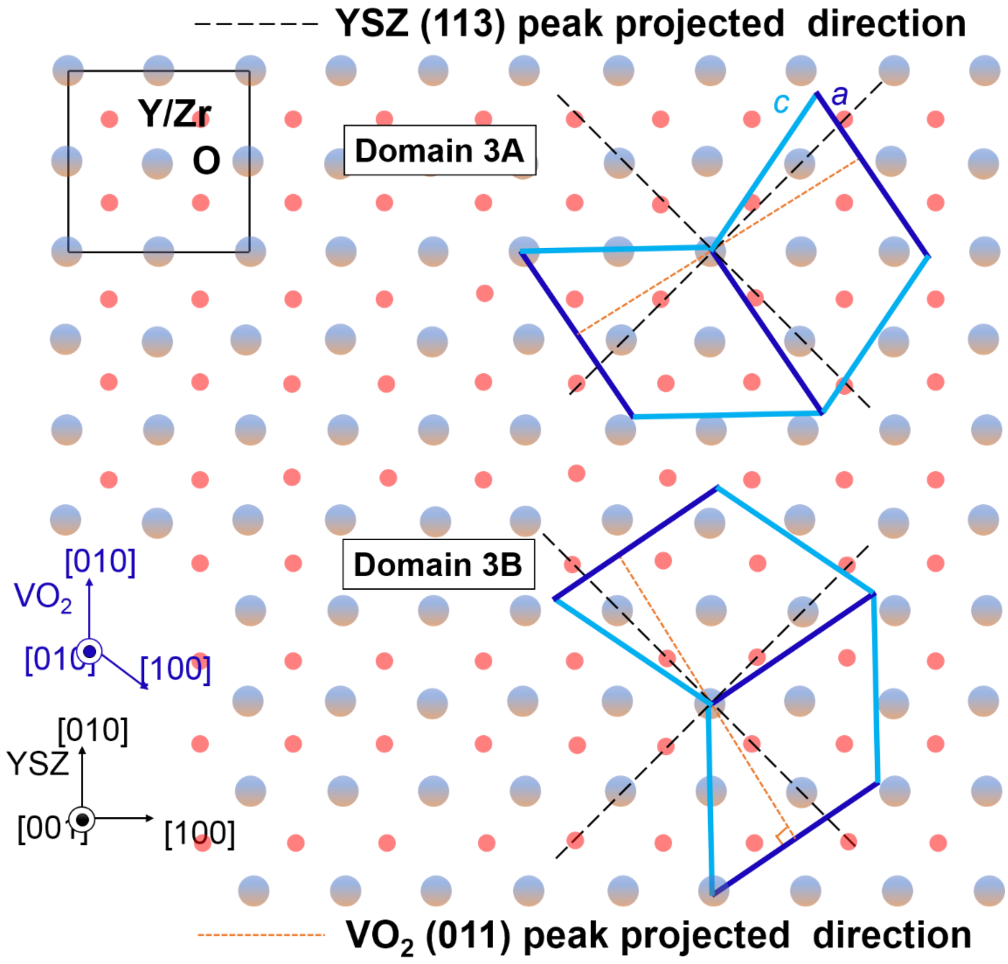

Figure S2. Domain configurations of Domain 3.

2. The Detailed Procedures for the Transport Measurements

In this work, we used Ecopia HMS-5000 Hall Measurement System (Bridge Technology Company) to obtain the resistivity, electron concentration and mobility. The controller interface is shown in Figure S3a. The sample stage with four probes and magnets is shown in Figure S3b. The four-probe technique was employed using van der Pauw method. The four probes first pressed on the Au/Ti electrodes. Then the transport properties were measured under the variable temperature in situ.

For the resistivity measurement, the magnets were moved out of the sample stage (~5000 Oe). In other words, the resistivity was measured without the application of magnetic field. However, for the electron mobility and concentration measurements, the magnets needed to be constantly moving back and forth. Consequently, the four probes pressed on the sample were displaced by the mechanical movement of the magnets, resulting in a large beating of the measured mobility and concentration data. Therefore, the displacement of the four probes during mobility and concentration measurements makes the data beating, while there is no beating of the resistivity data in the main text.

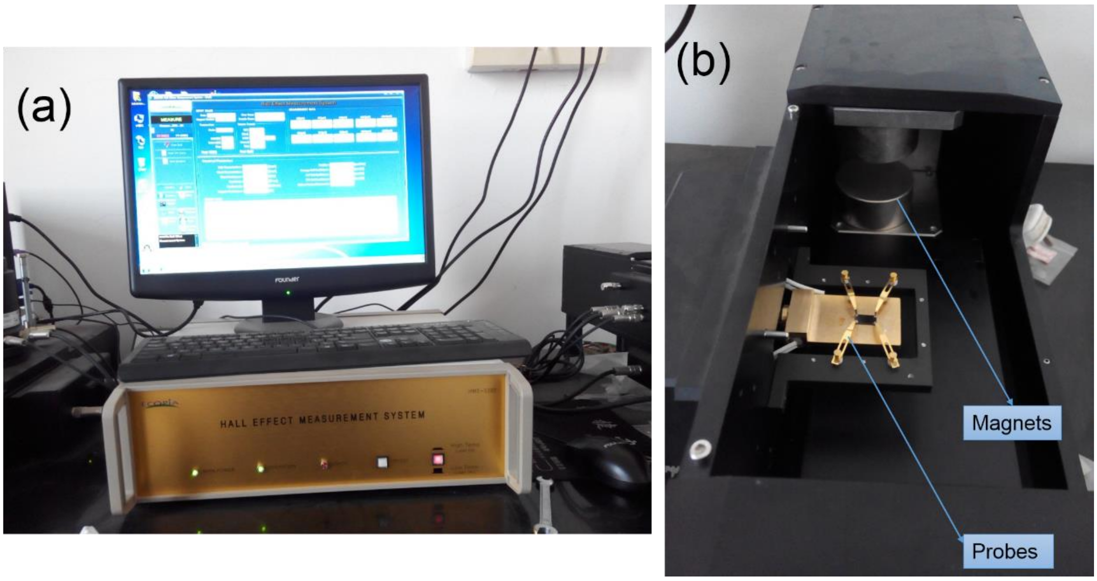

Figure S3. (**a**) Ecopia HMS-5000 Hall Measurement System; (**b**) The four-probe stage with movable magnets.
